# Enhanced Rupture Force in a Cut-Dispersed Double-Network Hydrogel

**DOI:** 10.3390/gels9020158

**Published:** 2023-02-16

**Authors:** Shilei Zhu, Dongdong Yan, Lin Chen, Yan Wang, Fengbo Zhu, Yanan Ye, Yong Zheng, Wenwen Yu, Qiang Zheng

**Affiliations:** 1 College of Physics, Taiyuan University of Technology, Taiyuan 030024, China; 2 College of Materials Science & Engineering, Taiyuan University of Technology, Taiyuan 030024, China; 3 Shanxi-Zheda Institute of Advanced Materials and Chemical Engineering, Taiyuan 030024, China; 4 Institute for Chemical Reaction Design and Discovery, Hokkaido University, Sapporo 001-0021, Japan; 5 Ministry of Education Key Laboratory of Macromolecular Synthesis and Functionalization, Department of Polymer Science and Engineering, Zhejiang University, Hangzhou 310027, China

**Keywords:** double-network hydrogels, cut dispersion, fracture, toughness

## Abstract

The Kirigami approach is an effective way to realize controllable deformation of intelligent materials via introducing cuts into bulk materials. For materials ranging from ordinary stiff materials such as glass, ceramics, and metals to soft materials, including ordinary hydrogels and elastomers, all of them are all sensitive to the presence of cuts, which usually act as defects to deteriorate mechanical properties. Herein, we study the influence of the cuts on the mechanical properties by introducing “dispersed macro-scale cuts” into a model tough double network (DN) hydrogel (named D-cut gel), which consists of a rigid and brittle first network and a ductile stretchable second network. For comparison, DN gels with “continuous cuts” having the same number of interconnected cuts (named C-cut gel) were chosen. The fracture tests of D-cut gel and C-cut gel with different cut patterns were performed. The fracture observation revealed that crack blunting occurred at each cut tip, and a large wrinkle-like zone was formed where the wrinkles were parallel to the propagation direction of the cut. By utilizing homemade circular polarizing optical systems, we found that introducing dispersed cuts increases the rupture force by homogenizing the stress around the crack tip surrounding every cut, which reduces stress concentration in one certain cut. We believe this work reveals the fracture mechanism of tough soft materials with a kirigami cut structure, which should guide the design of advanced soft and tough materials along this line.

## 1. Introduction

Morphing materials, including elastomers and gels, notable for their softness, high reversible stretchability, and functionalities [1] find a wide range of advanced technical applications, from biomedical adhesives [2,3,4,5,6], scaffolds for cell culture [7,8,9], and tissue engineering [10,11,12], to soft robotics [13,14,15,16,17] and stretchable electronics [18,19,20]. Among these materials, hydrogels have received increasing attention owing to their similarity to soft bio-tissues and multiresponsiveness to external stimuli. While as a kind of morphing material, these bulk hydrogels show a relatively small amplitude of deformation due to their geometric continuity. The Kirigami approach is an effective strategy by introducing cuts to disrupt the continuity to enhance the deformation ability [21,22,23,24,25]. However, given the intrinsic brittle nature of conventional hydrogels, the fabrication of gel with a kirigami structure that can sustain the force change during deformation remains a challenge.

Since the beginning of the 21st century, research on overcoming intrinsic hydrogel brittleness has progressed tremendously, and many great efforts have been made in fabricating strong and tough soft materials through chemical modification methods [1,26,27,28,29,30,31] (e.g., introducing sacrificial bonds [32,33,34,35,36]) or reinforcing fillers [37,38,39,40,41,42,43,44,45,46]. Taking the double-network (DN) strategy as a typical example, the DN hydrogels are strong and tough hydrogels consisting of two kinds of interpenetrating polymer networks with contrasting physical features: i.e., a stiff and brittle first network with dilute, densely crosslinked short chains and a soft and ductile second network with concentrated, loosely crosslinked long chains [35]. During deformation, the brittle first network breaks into clusters to dissipate a large amount of energy, while the stretchable second network keeps the integrity of the whole material without catastrophic failure [35]. The resultant strong and tough DN hydrogels, despite containing high water contents (~90 wt%), exhibit relatively high stiffness (elastic modulus of 0.1–1.0 MPa), high mechanical strength (nominal tensile stress at break *σ*_b_ of 1∼10 MPa, strain at break *ε*_b_ of 1000–2000%), and high toughness (fracture energy *Γ* of 10^3^–10^4^ J m^−2^) [35]. These mechanical performances are greatly superior to the SN gels of their individual components and even comparable to human load-bearing tissues and some tough industrial rubbers [32,35]. The double-network strategy has recently been extended to multiple-network elastomers exhibiting extremely reinforced mechanical strength and toughness without sacrificing stretchability [33,47,48,49].

On the basis of the aforementioned developments, tough hydrogels with kirigami structures were created. For example, a shape memory organohydrogel sheet cut with complex configurations can be transformed between distinct configurations by multistep shape memorization complemented with an external force [50,51,52]. The metal-coordinated tough hydrogels with complex geometries created by photolithographic polymerization can afford additional stretchability and better compliance to wrap on curved surfaces [21,53,54]. In addition, the tough composite gels incorporating a series of cutouts show a high degree of deformation freedom and large deformation amplitude of the responsive gel strips [22]. The above-mentioned tough hydrogels with kirigami structure show potential applications as flexible electronics, biomedical devices, and soft actuators.

**Figure 1:**
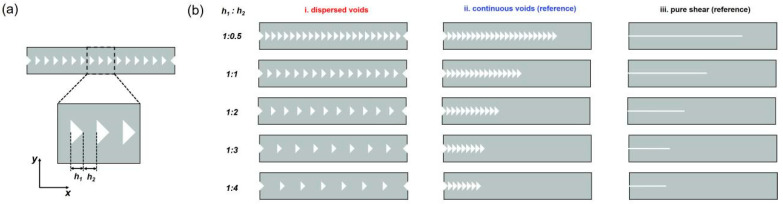
Scheme of the structural models with dispersed cuts and continuous cuts patterns for fracture experiments. (**a**) Scheme of the structural model containing an array of dispersed cuts investigated in this work. The length scale *h*_1_ is defined as the horizontal size of the triangle-shaped cuts, while *h*_2_ represents the horizontal spacing between adjacent cuts. In this work, the *h*_1_ is kept constant at 1.5 mm for simplicity. (**b**) The structural models with dispersed cuts at different spacing ratios *h*_1_:*h*_2_ (1:0.5, 1:1, 1:2, 1:3, and 1:4) and their counterpart structural models with the same number of aligned continuous cuts. The pure shear model was used as the reference.

The cuts introduced in the bulk gels generally act as defects in gels, which strongly affect the physical and mechanical properties [55]. However, very few studies have focused on the influence of introduced cuts in the kirigami structure on the mechanical properties of tough gels. In this work, the typical tough DN hydrogel is selected as a model system to study the effect of introduced cuts. The tough DN hydrogel is chosen for its unprecedented mechanical performance and easy synthesis among many hydrogel materials. It is known that DN hydrogels have rate-independent deformation behavior and negligible molecular interactions [56]. More importantly, the near-tip yielding region of DN hydrogels, where the deformation is so large and energy dissipation significantly occurs, can be directly observed by a birefringence observation experimental setup [56]. The dispersed cuts with a triangle shape were first introduced into the DN gels (named D-cut gels) to study the influence of cuts. For comparison, the DN gels with continuous cuts (named C-cut gel) and with pure shear geometry were also prepared (Figure 1). Combining the birefringence observation and the fracture tests, we found that the introduction of dispersed cuts can increase the rupture force by homogenizing the stress around the crack tip surrounding every cut, avoiding stress concentration in one certain cut. Around each cut, a large damage zone was formed to dissipate considerable energy to endow the material with toughness. We believe this work not only provides some insights into the fracture of tough soft materials with kirigami-cut structure but also guides the design of soft and tough materials with targeted mechanical properties by the introduction of kirigami cuts.

## 2. Results and Discussion

### 2.1. Effect of Dispersed Cuts on the Fracture Behavior of DN Hydrogel Specimens

A series of DN hydrogel specimens with dispersed and continuous cuts were created by a laser cutter (named D-cut gels and C-cut gels, respectively). Although the surface of the cross-section was burnt due to the high intensity of the laser, the fracture curve of DN gels cut with laser was almost overlapped with the gels cut by stainless steel blades, indicating that laser burns of cuts have negligible influence on the mechanical properties (Figure S1). This is due to how DN gels can form a large damage zone around the cuts during deformation [57], resulting in their robust mechanical properties, which are insensitive to the laser burns around the cuts. Figure 1 depicts the schematic illustration of the structural patterns containing an array of dispersed or continuous cuts. The length scale *h*_1_ is defined as the horizontal size of the triangle-shaped cuts, while *h*_2_ represents the horizontal spacing between adjacent triangle-shaped cuts. In this work, the size of *h*_1_ is kept constant at 1.5 mm for simplicity, and the spacing of *h*_2_ is tuned from 0.75 mm to 6.0 mm. According to the different spacing *h*_2_, the D-cut gel specimens were prepared from these different structural patterns with dispersed cuts at different spacing ratios *h*_1_:*h*_2_ (1:0.5, 1:1, 1:2, 1:3, and 1:4) and their counterpart C-cut specimens with aligned continuous cuts, as shown in Figure 1. Note that for comparison, the C-cut gel specimens are prepared to have the same number of aligned continuous cuts as the D-cut gel specimens such that the overall lengths of the uncracked ligaments can be kept the same between these specimens. The “continuous cuts” geometry in C-cut gels is more like “pure shear” geometry with a long crack and triangle-shaped crack front.

We first test the tensile behavior of the original DN hydrogel. As shown in Figure S2a, the original DN hydrogel (without cuts) shows the characteristic stress–stretch ratio curve with remarkable stress yielding (*σ*_y,tens_ = 0.57 MPa), which corresponds to the onset of necking (highly deformed region) in the tensile sample [56]. As elucidated in the previous study, the brittle first network is considered to severely rupture into fragments, and the stretchable second network is highly deformed in the necking region [35]. Such stress-yielding occurs ahead of the crack tip, reducing the stress concentration at the crack tip, thereby blunting the crack tip and enhancing the fracture resistance of the material [56].

**Figure 2:**
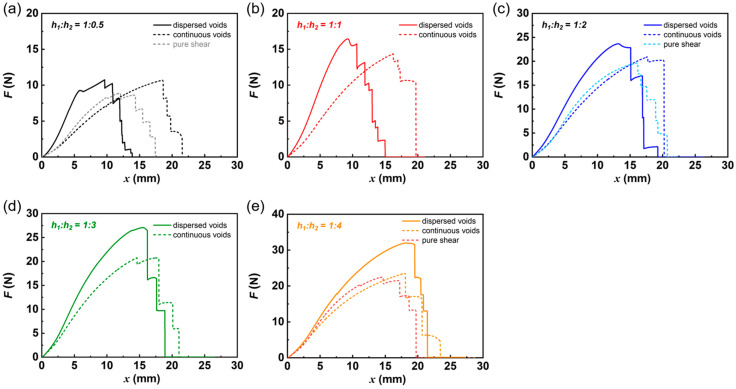
Representative force curves of highly deformable DN hydrogels under different structural models with dispersed cuts (D-cut gels) and continuous cuts (C-cut gels). The representative force (*F*)–displacement (*x*) curves of DN hydrogels under different structural models containing dispersed cuts and continuous cuts at different spacing ratios *h*_1_:*h*_2_ of 1:0.5 (**a**), 1:1 (**b**), 1:2 (**c**), 1:3 (**d**), and 1:4 (**e**). The force curves of samples with a pure shear geometry were also provided in (**a**,**c**,**e**) for comparison.

We next investigated fracture behavior in these patterned DN hydrogel specimens with dispersed and continuous cuts to demonstrate the effect of dispersed cuts (Figure S3). The D-cut and C-cut gel specimens were loaded in the vertical direction using displacement control at a constant displacement rate of 50 mm/min (the original length between clamps was kept constant at around 10 mm, corresponding to a stretch rate of 0.08 s^−1^). Compared with samples without cuts shown in Figure S2, the mechanical properties of the sample with cuts showed significant deterioration. Figure 2a–e further show the force (*F*)–displacement (*x*) curves of D-cut and C-cut DN hydrogel specimens during loading under different structural patterns, containing dispersed and continuous cuts at different spacing ratios *h*_1_:*h*_2_ of 1:0.5, 1:1, 1:2, 1:3, and 1:4. Here, we needed to emphasizes that the force–displacement curves of samples with C-cut and “pure shear” geometry are almost overlapped, indicating that the crack shape in the crack front has no obvious influence on the mechanical properties.

Regardless of spacing ratios *h*_1_:*h*_2_, the force curves of D-cut gel specimens exceed those of C-cut specimens from the initial stage of loading. Taking spacing ratio *h*_1_:*h*_2_ of 1:0.5 as an example, for the D-cut specimen, the force increases with increasing displacement between clamps until reaching a critical value (rupture force *F*_rupture_ = 10.84 ± 0.11 N occurring at *x*_rupture_ = 9.83 ± 0.38 mm), at which point the specimen begins to rupture, as seen in the snapshots during loading shown in birefringence experiment in the later section. A drop in load occurs during specimen rupture, as some regions sustain load ruptures to lose load-bearing capability. By contrast, for the C-cut specimen, the force increases in a relatively slow manner, with increasing displacement between clamps until reaching a critical value (rupture force *F*_rupture_ = 9.97 ± 0.60 N occurring at *x*_rupture_ = 15.73 ± 2.54 mm), at which point the specimen begins to rupture.

**Figure 3:**
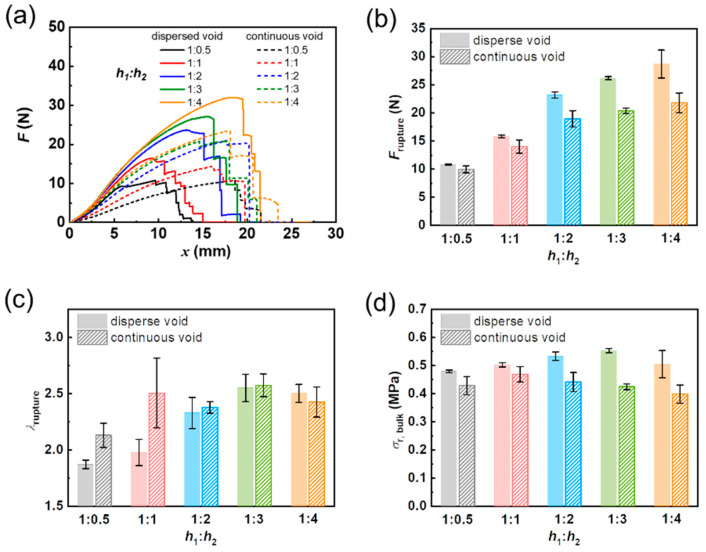
Effect of spacing ratios *h*_1_:*h*_2_ and dispersed/continuous cuts on the mechanical behaviors of DN hydrogel samples in fracture experiments. (**a**) Summarized force (*F*)–displacement (*x*) curves of DN hydrogels. (**b**) Rupture force (*F*_rupture_), (**c**) critical stretch ratio at rupture point (*λ*_rupture_), and (**d**) critical bulk stress at rupture point (*σ*_r,bulk_) for DN hydrogel samples containing dispersed cuts (D-cut gels) and continuous cuts (C-cut gels) at different spacing ratios *h*_1_:*h*_2_ (1:0.5, 1:1, 1:2, 1:3, and 1:4).

Figure 3a summarizes all the representative force–displacement curves of DN hydrogel specimens with different structural patterns containing dispersed cuts and continuous cuts at different spacing ratios *h*_1_:*h*_2_ of 1:0.5, 1:1, 1:2, 1:3, and 1:4. For the D-cut specimens, by increasing the horizontal spacing between adjacent triangle-shaped cuts *h*_2_, both the critical rupture force *F*_rupture_ and critical displacement *x*_rupture_ increase (Figure 3a). This is reasonable because the cross-sectional area of the uncracked ligament in the specimens that sustain the load increases with the spacing between adjacent cuts *h*_2_, giving rise to a large load-bearing capability.

We next analyzed the critical rupture force *F*_rupture_ in Figure 3b. It is also found that regardless of different spacing ratios *h*_1_:*h*_2_, the critical rupture forces *F*_rupture_ of the D-cut specimens are higher than these of the C-cut specimens, and the deviation between *F*_rupture_ of the D-cut specimens and C-cut specimens increases with *h*_2_ (Figure 3b). Specifically, in the case of spacing ratio *h*_1_:*h*_2_ of 1:0.5, the D-cut specimens show a critical rupture force *F*_rupture_ of 10.84 ± 0.11 N, slightly higher than that of the C-cut specimens (rupture force *F*_rupture_ = 9.97 ± 0.60 N). While in the case of spacing ratio *h*_1_:*h*_2_ of 1:4, the D-cut specimens demonstrate the critical rupture force *F*_rupture_ of 28.69 ± 2.48 N, much higher than that of the C-cut specimens (rupture force *F*_rupture_ = 21.79 ± 1.76 N). Note that at the same spacing ratios *h*_1_:*h*_2_, the D-cut specimens and C-cut specimens all have the same cross-sectional area of the uncracked ligament (the overall cross-sectional area of the whole specimen subtracted by the area occupied by cuts). Because the C-cut geometry is more like the conventional “pure shear” geometry with a precut, this result also indicates that the dispersed cuts can produce an enhanced rupture force than the pure shear specimen with a precut.

The critical stretch ratio at the rupture point *λ*_rupture_ is further analyzed in Figure 3c. In the case of spacing ratio *h*_1_:*h*_2_ of 1:0.5, the C-cut specimens show a much higher critical stretch ratio at rupture point *λ*_rupture_ (= 1.87 ± 0.04) than that of the D-cut specimens (*λ*_rupture_ = 2.13 ± 0.11). With further increasing spacing *h*_2_ until reaching spacing ratio *h*_1_:*h*_2_ of 1:2, the D-cut specimens begin to exhibit comparable *λ*_rupture_ with these of the C-cut specimens. In particular, at a spacing ratio *h*_1_:*h*_2_ of 1:4, the D-cut specimens exhibit *λ*_rupture_ of 2.50 ± 0.08, while the C-cut specimens show *λ*_rupture_ of 2.43 ± 0.13.

We next compare the critical bulk stress at the rupture point (*σ*_r,bulk_) of different specimens. We should note here that critical bulk stress at the rupture point (*σ*_r, bulk_) is defined as the rupture force *F*_rupture_ divided by the cross-sectional area of the uncracked ligament (the overall cross-sectional area of the whole specimen subtracted by the area occupied by cuts). It is clearly shown in Figure 3d that the *σ*_r,bulk_ of all the samples is located in the narrow range of 0.40 MPa to 0.55 MPa, which is close to the yielding stress of DN hydrogel (*σ*_y,tens_ = 0.57 MPa). Additionally, it is found that regardless of spacing ratios *h*_1_:*h*_2_, the D-cut specimens show higher *σ*_r,bulk_ than the C-cut specimens.

**Figure 4:**
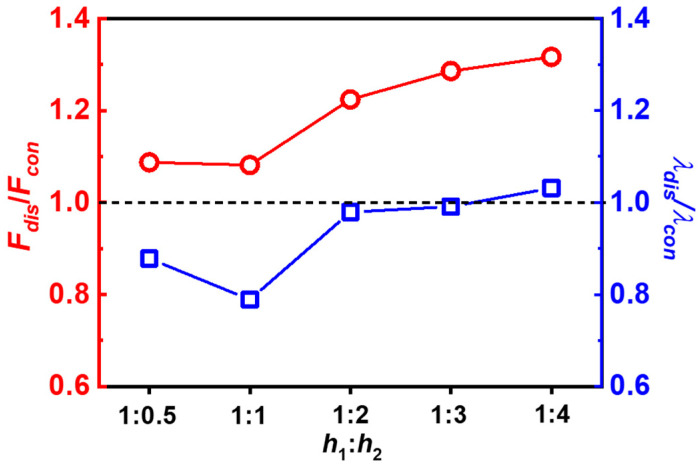
The rupture force ratio (*F*_dis_/*F*_con_) and normalized rupture stretch ratio (*λ*_dis_/*λ*_con_) as functions of spacing ratios *h*_1_:*h*_2_. The *F*_dis_ and *F*_con_ represent the rupture forces in fracture specimens containing dispersed cuts and continuous cuts, respectively. The *λ*_dis_ and *λ*_con_ denote the rupture stretch ratios in fracture experiments containing dispersed cuts (D-cut gels) and continuous cuts (C-cut gels), respectively.

To demonstrate the effect of dispersed cuts, we then plot the rupture force ratio (*F*_dis_/*F*_con_) and normalized rupture stretch ratio (*λ*_dis_/*λ*_con_) as functions of spacing ratios *h*_1_:*h*_2_ in Figure 4. Here, the *F*_dis_ and *F*_con_ represent the rupture forces in fracture specimens containing dispersed cuts and continuous cuts at the same spacing ratios *h*_1_:*h*_2_, respectively. The *λ*_dis_ and *λ*_con_ denote the rupture stretch ratios in fracture experiments containing dispersed cuts and continuous cuts at the same spacing ratios *h*_1_:*h*_2_, respectively. The rupture force ratio *F*_dis_/*F*_con_ also can be seen as the enhancement ratio of rupture force of fracture specimens by the dispersed cuts. As shown in Figure 4, the enhancement ratio increases from 1.08 to 1.32 with changing spacing ratios *h*_1_:*h*_2_ from 1:0.5 to 1:4. In the meantime, the normalized rupture stretch ratio *λ*_dis_/*λ*_con_ increases from 0.8 to 1.03 with changing spacing ratios *h*_1_:*h*_2_ from 1:0.5 to 1:4. This suggests that by increasing spacing *h*_2_, the dispersed cuts can enhance the rupture force without sacrificing the stretchability of the bulk materials, thereby increasing the fracture resistance.

### 2.2. Birefringence Observation on the Rupture of Specimens Containing Dispersed Cuts and Continuous Cuts

We next try to understand why the dispersed cuts give rise to the enhanced rupture force. We perform the birefringence observation on the rupture of specimens containing dispersed cuts and continuous cuts using a birefringence observation experimental setup, shown in Figure S1; the specimen is placed between two crossed circular polarized films, and the two films were placed between a white lamp and a video camera [56]. Real-time imaging of the birefringence for the specimens during loading allows us to investigate the crack tip behaviors at the rupture point for the D-cut and C-cut specimens. By this means, the highly deformed region of the specimens during rupture can be observed.

**Figure 5:**
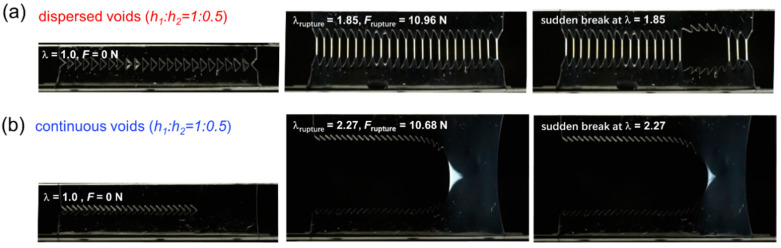
Birefringence observation during fracture of D-cut and C-cut gels at spacing ratio *h*_1_:*h*_2_ of 1:0.5. (**a**,**b**) The representative snapshots of crack evolution in DN hydrogel samples under different structural models containing dispersed cuts ((**a**) D-cut gel) and continuous cuts ((**b**) C-cut gel) at spacing ratio *h*_1_:*h*_2_ of 1:0.5.

**Figure 6:**
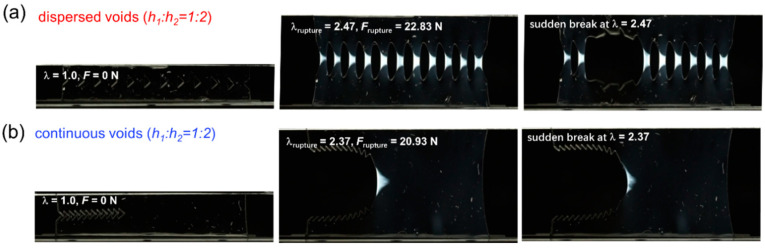
Birefringence observation during fracture of D-cut and C-cut gels at spacing ratio *h*_1_:*h*_2_ of 1:2. (**a**,**b**) The representative snapshots of crack evolution in DN hydrogel samples under different structural models containing dispersed cuts ((**a**) D-cut gel) and continuous cuts ((**b**) C-cut gel) at spacing ratio *h*_1_:*h*_2_ of 1:2.

**Figure 7:**
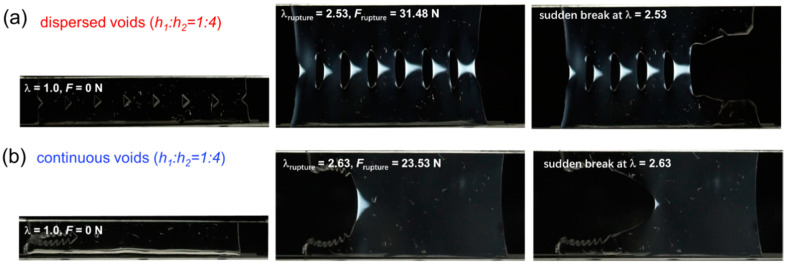
Birefringence observation during fracture of D-cut and C-cut gels at spacing ratio *h*_1_:*h*_2_ of 1:4. (**a**,**b**) The representative snapshots of crack evolution in DN hydrogel samples under different structural models containing dispersed cuts ((**a**) D-cut gel) and continuous cuts ((**b**) C-cut gel) at spacing ratio *h*_1_:*h*_2_ of 1:4.

Figure 5, Figure 6 and Figure 7 display the representative snapshots of crack evolution in DN hydrogel specimens under different structural patterns containing dispersed cuts and continuous cuts at spacing ratios *h*_1_:*h*_2_ of 1:0.5, 1:2, and 1:4, respectively. As seen in Figure 5a, in the case of spacing ratio *h*_1_:*h*_2_ of 1:0.5, for the D-cut specimens, owing to the small spacing *h*_2_ between adjacent cuts, all the spacing regions are homogeneously deformed to a high level to exhibit a strong birefringence at the rupture point, just like the strong birefringence observed in the tensile process as reported in the previous study by Gong et al. After reaching the critical value of rupture force, some highly deformed spacing regions suddenly rupture, as seen in Figure 5a. While in the case of C-cut specimens, a large bright triangle-shaped birefringence region occurs ahead of the crack tip (Figure 5b), like that observed in the “pure shear” geometry. As elucidated in the previous work by Gong et al., such a large bright birefringence area corresponds to the stress-yielding occurring ahead of the crack tip accompanied by the formation of a large yielding zone, where the brittle first network is considered to severely rupture into fragments and the stretchable second network is highly deformed. The formation of a large yielding zone reduces the stress concentration at the crack tip, thereby blunting the crack tip and enhancing the fracture resistance of the material [56].

With increasing spacing *h*_2_ between adjacent cuts to reach a spacing ratio *h*_1_:*h*_2_ of 1:2, the spacing regions of the D-cut specimens are highly deformed to display nearly trapezoid-shaped birefringence areas, each of which is smaller than the triangle-shaped birefringence area in the C-cut specimens (Figure 6). With further increasing of spacing *h*_2_ between adjacent cuts to reach a spacing ratio *h*_1_:*h*_2_ of 1:4, the spacing regions of the D-cut specimens are highly deformed to display nearly triangle-shaped birefringence areas, some of which are comparable to the triangle-shaped birefringence area in the C-cut specimens (Figure 7). Additionally, it should be mentioned that the C-cut specimens all exhibit the directed crack propagation direction from left to right like the conventional “pure shear” specimen, while the D-cut specimens rupture randomly in the weakest points of the spacing regions.

**Figure 8:**
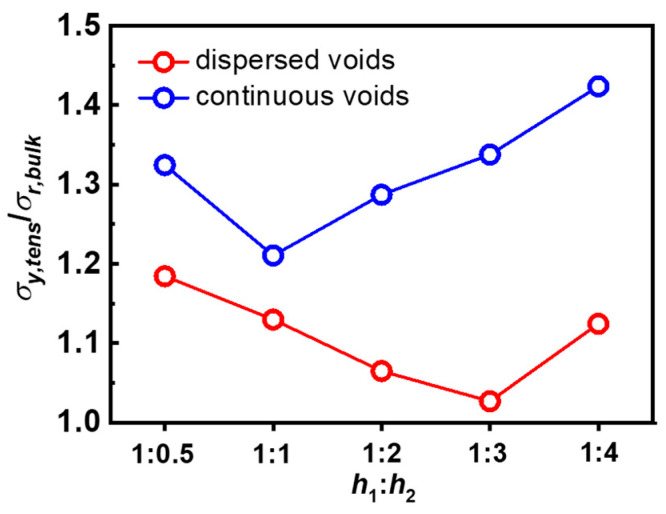
The normalized stress concentration ratio (the ratio between tensile yielding stress and critical bulk stress, *σ*_y,tens_*/σ*_r,bulk_) as functions of spacing ratios *h*_1_:*h*_2_.

It is well known that the specimen ruptures only when the material point ahead of the crack tip (or ahead of cuts) is highly stretched to possess stress *σ*_tip_ exceeding the critical threshold value *σ*_threshold_. Owing to the stress concentration, the crack tip stress *σ*_tip_ is usually amplified from the bulk stress *σ*_bulk_. If we denote a stress concentration factor α as the ratio of crack tip stress *σ*_tip_ over the bulk stress *σ*_bulk_, $\sigma_{tip}=\alpha \sigma_{bulk}$, a severe stress concentration in the crack tip means that the crack tip stress *σ*_tip_ will be amplified to α times the bulk stress *σ*_bulk_. Because the yielding dominates the yielding zone area (birefringence area) ahead of the crack tip, we can simply consider that the maximum stress of the material point ahead of the crack tip, which is also the critical threshold value *σ*_threshold_, should be related to yielding stress *σ*_y,tens_ by a factor of β; thus we have $\sigma_{threshold}=\beta \sigma_{y,tens}$. For the same material, β should be a constant. Then, the rupture criterion will be $\sigma_{tip}\geq \sigma_{threshold}$, thus $\alpha \sigma_{bulk}\geq \beta \sigma_{y,tens}$. With increasing the bulk stress until reaching the critical bulk stress $\sigma_{r,\;bulk}$ at the rupture point, the rupture occurs. So, we have $\alpha \sigma_{r,bulk}=\beta \sigma_{y,tens}$; thus, a normalized stress concentration factor will be $\frac{\alpha }{\beta }=\frac{\sigma_{y,tens}}{\sigma_{r,bulk}}$. We next plot the $\frac{\sigma_{y,tens}}{\sigma_{r,bulk}}$ for D-cut specimens and C-cut specimens in Figure 8. It can be seen that the values of $\frac{\sigma_{y,tens}}{\sigma_{r,bulk}}$ for the D-cut specimens are lower than those of the C-cut specimens, suggesting that the stress concentration due to the crack tip (or cuts) is less severe in the “dispersed cuts” cases. Note that for common DN hydrogels, even in the “continuous cuts” cases, the formation of a large yielding zone already remarkably reduces the stress concentration at the crack tip, thereby blunting the crack tip and enhancing the fracture resistance of the material. Here, we show that the introduction of a “dispersed cuts” pattern can further reduce the stress concentration at the crack tip, enhancing the rupture force. It can also be observed from the birefringence snapshots for D-cut specimens in Figure 5, Figure 6 and Figure 7 that the spacing regions between every two adjacent cuts, which are sustaining loads, are all deformed to a high level ahead of the crack tip, meaning that the dispersed cuts may homogenize the stress around the crack tip surrounding every cut, avoiding stress concentration in one certain cut. Therefore, the “dispersed cuts” specimens can sustain an enhanced rupture force.

### 2.3. Characteristic Fracture Structure of the DN Gels with Dispersed and Continuous Cuts

**Figure 9:**
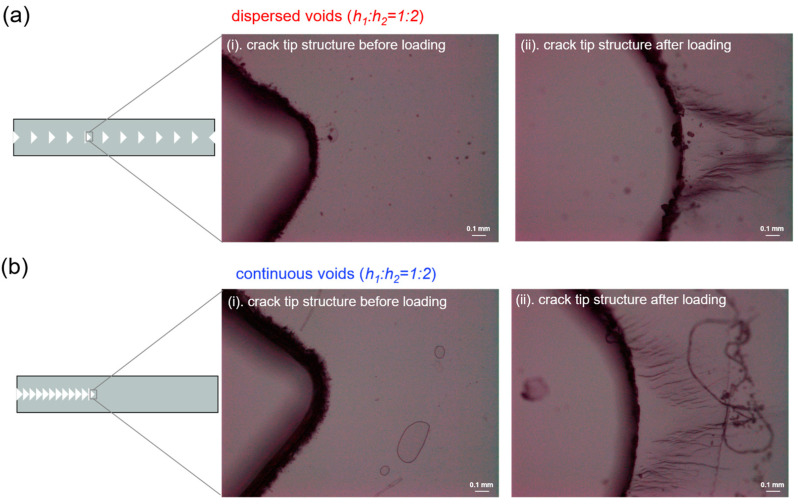
The crack tip structure of DN hydrogels was observed by optical microscopy. (**a**,**b**) The representative crack tip structure in DN hydrogel samples before and after loading to a stretch ratio λ of 2 under different structural models containing dispersed cuts (**a**) and continuous cuts (**b**) at spacing ratio *h*_1_:*h*_2_ of 1:2. Wrinkles-like damaged structure can be observed ahead of the crack tip, corresponding to the damage zone observed by birefringence.

To clarify the fracture structure in the gels with cuts, we further observed the characteristic fracture structure of gels by optical microscopy, and the results are presented in Figure 9. These images were obtained before and after stretching for λ = 2.1. Before stretching, a smooth surface was observed around the crack tip, and there was no characteristic structure under the microscopic scale (Figure 9a(i),b(i)). This is in agreement with the fact that without deformation, the PAMPS network remains undamaged. After stretching the gel for λ = 2.1, we found that the deformation can induce a wrinkle-like fracture structure formed around the crack tips. For the D-cut gel with dispersed cuts, a wrinkle-like fracture structure was observed around every cut. While for the C-cut gel with continuous cuts, only the most inside cut (marked in Figure 9b) shows a wrinkle-like fracture structure. The characteristic direction of the wrinkles is vertical to the direction of the applied force, suggesting a parallel and gradient fracture of the first network PAMPS along the stretching direction [57]. The above wrinkle structure also has been observed in the ultrathin film DN gels with 100 μm thickness [57]. The wrinkle structure region around the crack tip was considered as the damage zone dissipating a large amount of energy. The D-cut gels can form several damage zones while the C-cut gels can only form one damage zone; thus, the D-cut gels possibly dissipate more energy compared to the C-cut gels.

## 3. Conclusions

We studied the fracture behaviors of tough materials with a kirigami-cut structure. By incorporating dispersed cuts into a tough hydrogel matrix, it can effectively improve the force at the rupture point. We demonstrate the effect of dispersed cuts by comparing the fracture behavior of D-cut specimens with dispersed cuts with that of the C-cut specimens having the same number of continuous cuts. By combining fracture test and fracture observation, we find that the introduction of dispersed cuts increases the rupture force by homogenizing the stress around the crack tip surrounding every cut, avoiding stress concentration in one certain cut. The quantitative analysis of a normalized stress concentration factor $\frac{\sigma_{y,tens}}{\sigma_{r,bulk}}$ supports the finding that the introduction of a “dispersed cuts” pattern can reduce the stress concentration at the crack tip, enhancing the rupture force. We believe this work not only provides some insights into the fracture of tough soft materials with a kirigami-cut structure, but also guides the design of soft and tough materials with targeted mechanical properties by the introduction of kirigami cuts.

## 4. Materials and Methods

### 4.1. Materials

2-Acrylamido-2-methylpropanesulfonic acid sodium salt (NaAMPS), acrylamide (AAm), *N*, *N*′-methylenebis(acrylamide) (MBAA), and *α*-ketoglutaric acid (*α*-keto) were purchased at Shanghai Aladdin Bio-Chem Technology Co., Ltd. (Shanghai, China) and used as received. Milli-Q water (resistivity: 18.3 MΩ·cm) was used in all experiments.

### 4.2. Synthesis of DN Hydrogels

The poly(2-acrylamido-2-methylpropanesulfonic acid sodium salt)/polyacrylamide (PNaAMPS/PAAm) DN hydrogels were synthesized by a two-step sequential network formation technique following the literature [58]. The first PNaAMPS network of the DN hydrogels was synthesized from an aqueous solution of 1.0 M NaAMPS containing 3 mol% crosslinking agent, MBAA, and 1 mol% initiator, *α*-keto. To perform the polymerization, the solution was purged in an argon atmosphere to remove dissolved oxygen and then poured into a reaction cell consisting of a pair of glass plates with 0.5 mm spacing. The reaction cell was irradiated with UV light (365 nm) for 8 h. These gels (first network) were then immersed in an aqueous solution of 2.0 M AAm, containing 0.01 mol% MBAA and 0.01 mol% *α*-keto, for one day until swelling equilibrium was reached. The polymerization was performed again by 365 nm UV irradiation for 8 h. The as-prepared DN gels were then immersed in pure water to reach equilibrium to obtain the DN gels for further experiments.

### 4.3. Preparation of Various Cuts Patterns in DN Hydrogel Specimens

To prepare various cut patterns in DN hydrogels for fracture experiments, as illustrated in Figure 1, the DN hydrogel samples were cut using a laser cutter machine (ULTRA R5000, Universal Laser Systems, Inc., Yokohama, Japan). The length scale *h*_1_ is defined as the horizontal size of the triangle-shaped cuts, while *h*_2_ represents the horizontal spacing between adjacent cuts. The *h*_1_ is kept constant at 1.5 mm in this work, and the spacing *h*_2_ is controlled in various lengths of 0.75 mm, 1.5 mm, 3.0 mm, and 6.0 mm, respectively. Accordingly, the DN hydrogel specimens with structural cut patterns were prepared at different spacing ratios *h*_1_:*h*_2_ (1:0.5, 1:1, 1:2, 1:3, and 1:4).

### 4.4. Real-Time Birefringence Observation on the Fracture Tests

The experimental setup and sample size used for real-time birefringence observation of crack propagation in DN hydrogel specimens during fracture test are illustrated in Figure S1 [56]. The D-cut specimens and C-cut specimens were fixed with the pure shear clamps of a tensile tester machine (4466, Instron Instruments, Inc., Norwood, MA, USA), where the initial clamp distance was fixed at ~10 mm. The clamps were stretched by a tensile tester machine at a constant velocity of 50 mm/min, and the force–displacement curve was recorded. To perform real-time observation of the sample, the sample is placed in between two crossed circular polarized films. One polarizing film was set in front of a white lamp across the light path, and the other one was placed in front of the recording video camera. The in situ fracture process was recorded using an ordinary video camera (24 frames/s, 1920 × 1080 pixels, Sony α7S E-mount Camera, Sony Electronics Inc., San Diego, CA, USA). The entire procedure was performed in a dark room.

### 4.5. Tensile Test

The tensile mechanical properties of the DN hydrogels were measured with a commercial test machine (4466, Instron Instruments, Inc., USA.) in the air. The samples were cut into dumbbell shapes standardized as JISK6251-7 size (gauge length 12 mm, width 2 mm) with a gel cutting machine (Dumbbell Co., Ltd., Kawagoe, Japan). The nominal stress σ-stretch ratio *λ* curves were recorded while the sample gels were stretched at a constant velocity of 100 mm/min (strain rate of 0.14 s^−1^).

### 4.6. Microscopic Observation of Cuts in the DN Gels

Samples for the microscopic observation of tensile-induced fracture were shaped the same as shown in Figure 1. The samples were first experienced to a designated pre-strain λ = 2.1 under the tensile velocity of 50 mm/min and then taken out for the microscopic observation with a 5-folds objective lens at the free-standing state of the samples. For comparison, the gel without stretching was also observed.

## Data Availability

The data presented in this study are available on request from the corresponding author.

## Supplementary Materials

The following supporting information can be downloaded at https://www.mdpi.com/article/10.3390/gels9020158/s1, Figure S1: The cut surface and the pure shear fracture behaviors of DN hydrogels; Figure S2: The stress-strain curve of the bulk DN gels; Figure S3: Representative strain-stress curves of highly deformable DN hydrogels under different structural models with dispersed cuts and continuous cuts; Figure S4: Schematic diagram of birefringence observation set-up for fracture tests.
