# Supplementary material for: Enhanced Rupture Force in a Cut-Dispersed Double-Network Hydrogel
Source: Gels. 2023 Feb 16;9(2):158. doi: 10.3390/gels9020158 (PMC9956972; doi:10.3390/gels9020158)
Supplement: Supplementary file 1 [file gels-09-00158-s001.zip › gels-2180163-supplementary.pdf]

Supporting Information for

Enhanced Rupture Force in a Cut-Dispersed Double-Network Hydrogel

Shilei Zhu [1,†], Dongdong Yan [2,†], Lin Chen [2], Yan Wang [2], Fengbo Zhu [2,3], Yanan Ye [2,3,*], Yong Zheng [4,*], Wenwen Yu [2,3] and Qiang Zheng [2,5]

[1] College of Physics, Taiyuan University of Technology, Taiyuan 030024, China
[2] College of Materials Science & Engineering, Taiyuan University of Technology, Taiyuan 030024, China
[3] Shanxi-Zheda Institute of Advanced Materials and Chemical Engineering, Taiyuan 030024, China
[4] Institute for Chemical Reaction Design and Discovery, Hokkaido University, Sapporo 001-0021, Japan
[5] Ministry of Education Key Laboratory of Macromolecular Synthesis and Functionalization, Department of Polymer Science and Engineering, Zhejiang University, Hangzhou 310027, China
* Correspondence: yeyanan@tyut.edu.cn (Y.Y.); zhengyong@sci.hokudai.ac.jp (Y.Z.)
† These authors contributed equally to this work.

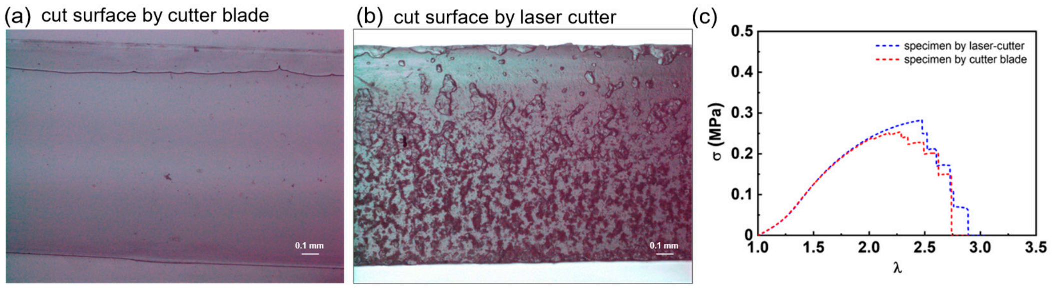

Figure S1. The cut surface and the pure shear fracture behaviors of DN hydrogels. (a) The cut surface of DN hydrogel by cutter blade. (b) The cut surface of DN hydrogel by laser cutter. (c) The mechanical comparison The pure shear fracture behaviors of DN hydrogel are hardly affected by the cut methods.

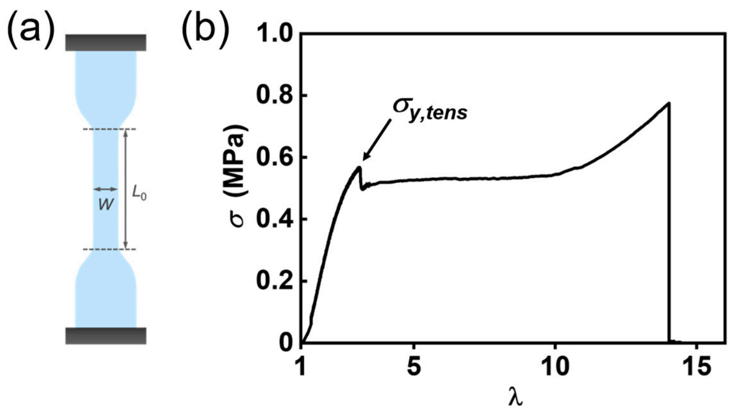

Figure S2 The stress-strain curve of the bulk DN gels. (a) Schematic diagram of the sample. The total length sample is 25 mm, the gauge length L_0 is 12 mm, and the width w is 2 mm. (b) The strain-stress curve of gel under uniaxial tensile deformation of bulk DN gels.

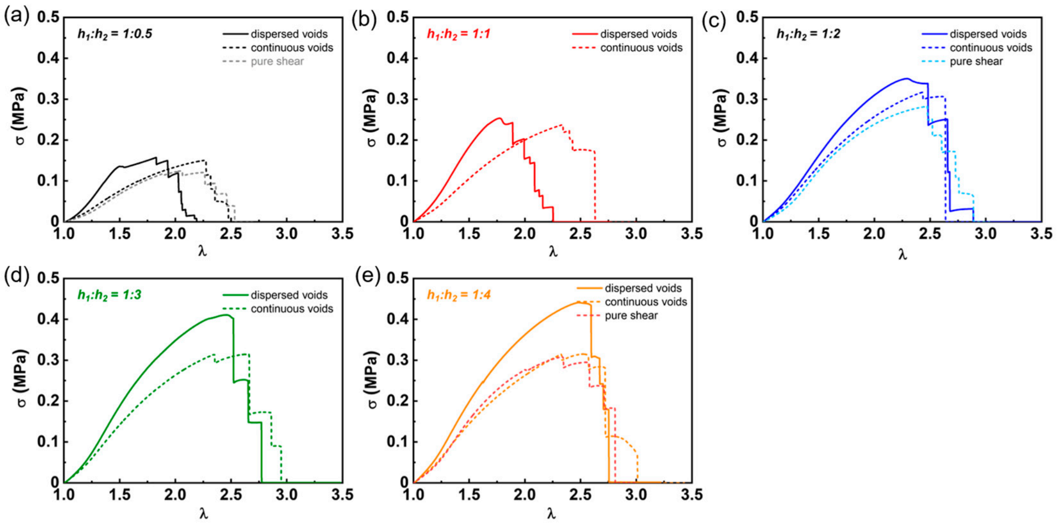

Figure S3. Representative strain-stress curves of highly deformable DN hydrogels under different

structural models with dispersed cuts and continuous cuts. The representative stress (MPa)-strain (λ) curves of DN hydrogels under different structural models containing dispersed cuts and continuous cuts at different spacing ratios h1: h2 of 1:0.5 (a), 1:1 (b), 1:2 (c), 1:3 (d) and 1:4 (e). The force curves of samples with a pure shear geometry were also provided in (a), (c), and (e) for comparison.

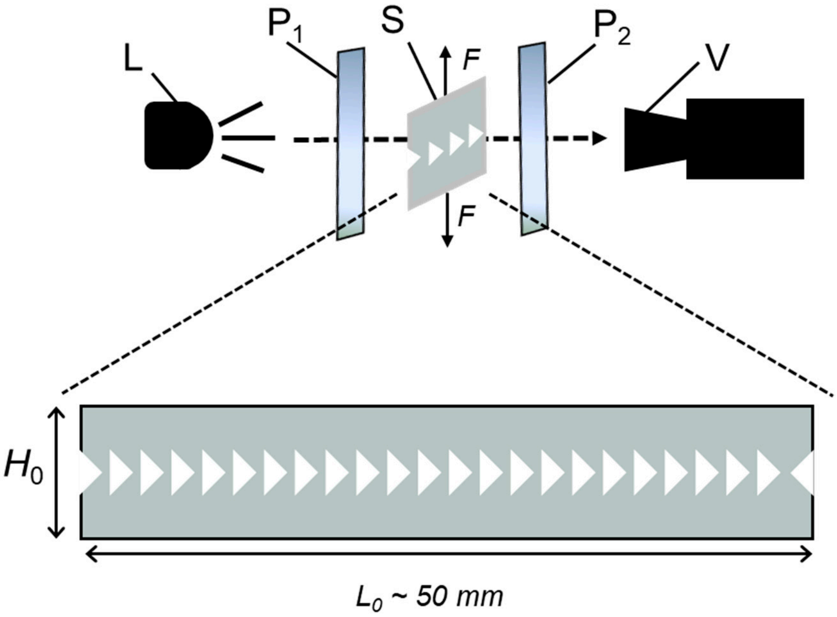

Figure S4. Schematic diagram of birefringence observation set-up for fracture tests. L: lamp, P_1, P_2: crossed circular polarized film, S: sample, V: a video camera. The sample was stretching in the direction shown by arrows.
